# Computational models in plant-pathogen interactions: the case of *Phytophthora infestans*

**DOI:** 10.1186/1742-4682-6-24

**Published:** 2009-11-12

**Authors:** Andrés Pinzón, Emiliano Barreto, Adriana Bernal, Luke Achenie, Andres F González Barrios, Raúl Isea, Silvia Restrepo

**Affiliations:** 1 Mycology and Phytopathology Laboratory, Department of Biological Sciences, Universidad de los Andes, Bogotá, Colombia; 2 Bioinformatics center, Colombian EMBnet node, Biotechnology Institute, National University of Colombia, Bogotá, Colombia; 3 Department of Chemical Engineering, Virginia Polytechnic Institute and State University, Blacksburg Virginia, USA; 4 Grupo de Diseño de Productos y Procesos, Department of Chemical Engineering, Los Andes University, Bogotá, Colombia; 5 Fundación IDEA, Centro de Biociencias, Hoyo de la puerta, Baruta 1080, Venezuela

## Abstract

**Background:**

*Phytophthora infestans *is a devastating oomycete pathogen of potato production worldwide. This review explores the use of computational models for studying the molecular interactions between *P. infestans *and one of its hosts, *Solanum tuberosum*.

**Modeling and conclusion:**

Deterministic logistics models have been widely used to study pathogenicity mechanisms since the early 1950s, and have focused on processes at higher biological resolution levels. In recent years, owing to the availability of high throughput biological data and computational resources, interest in stochastic modeling of plant-pathogen interactions has grown. Stochastic models better reflect the behavior of biological systems. Most modern approaches to plant pathology modeling require molecular kinetics information. Unfortunately, this information is not available for many plant pathogens, including *P. infestans*. Boolean formalism has compensated for the lack of kinetics; this is especially the case where comparative genomics, protein-protein interactions and differential gene expression are the most common data resources.

## Background

Control and management of plant diseases and the identification of factors that contribute to the spread a given plant pathogen attack are at the basis of phytopathology. Mathematical models and computational simulations have been used, along with molecular and physiological approaches, to solve these and other issues.

In the early 1990s the use of stochastic models in plant pathology was reviewed [1,2], mostly focused on epidemics. In this work we update topics not fully covered in previous reviews as well as associated experimental approaches that characterize the systems biology era [3]. Most of the review will focus on the *Phytophthora infestans *- *Solanum tuberosum *pathosystem, but its discussion will be general enough as to be applicable to any other plant pathogen system. A brief discussion of boolean networks and how this approach could drive the modeling of the compatible interaction between *P. infestans *and *S. tuberosum *is also introduced.

### Experimental approaches to the study of molecular plant-pathogen interactions in *Phytophthora *species

Plants use various strategies to resist infection by a particular pathogen [4]. These strategies are part of the plant's innate immune system and can be grouped into two broad categories [5]. The first recognizes common pathogen-associated molecular patterns (PAMPs), and acts as an early plant warning to potential infection [6]. This recognition leads to the induction of a basal plant defense, which in some cases includes a hypersensitive response (HR). The HR is characterized by the rapid death of cells surrounding the infected region and commonly leads to a broad spectrum plant response, the Systemic Acquired Resistance [7].

A second defense system in plants involves pairs of gene products, an effector molecule from the pathogen and an associated resistance protein (R) from the host, which recognizes it. This defense mechanism is highly specific and is triggered once a given effector is recognized by its associated *R *defense protein [5].

Plants with the capacity for protection from a pathogen attack are considered as resistants and a pathogen that lacks the ability to infect it is referred to as avirulent on that plant [4]. In this case, the host-pathogen interaction is considered incompatible. On the other hand, when a compatible interaction occurs, the pathogen becomes virulent and a plant that is incapable of resisting the attack is considered non-resistant.

Plant pathogens have developed several strategies to evade such plant defense responses and to become virulent. For some of these pathogens the evasion mechanisms are at least partially known, as in the case of bacteria such as *Pseudomonas syringae*. However, for most plant pathogen species, these evasion mechanisms are almost completely unknown. This is the case for *P. infestans*, the causal agent of *late blight of potato*, a disease that affects *S. tuberosum *and some other species in the *Solanaceae *family [8]. Oomycetes from the genus *Phytophthora *are plant pathogens devastating for agriculture and natural ecosystems [9]. For instance, in the United States alone, *P. infestans *causes estimated losses that exceed $US 5 billion annually [10].

Despite its economic importance, the fundamental molecular mechanisms underlying the pathogenicity of *P. infestans *are poorly understood. It was not until recent years that information crucial to the understanding of its genomics and infectious mechanisms was accessible to the research community [11]. For example, in 2006, the first effort to classify the secretome of plant pathogenic Oomycetes was carried out by Kamoun et al. Furthermore, although the general molecular events associated with the interaction between *P. infestans *and *S. tuberosum *were already known in 1991 [12], it was not until last year (2008) that all the known molecular and cytological processes underlying plant-pathogen interactions in various *Phytophthora *species were revised [9].

From the biological strategies used so far to study the processes underlying plant-pathogen interactions, three are most suitable as basis for a computational systems biology approach: (a) gene expression, (b) structural and comparative genomics and (c) protein-protein interactions.

### Gene expression

Gene expression approaches constitute a starting point from which to determine the best strategy for building a computational model of a plant disease. Host-expressed molecules give insights into the underlying defense mechanisms, whereas identification of the pathogen counterparts allows us to ascertain possible mechanisms of attack and/or avoidance mechanisms used to establish a disease.

#### Differential expression of particular genes

A common strategy in gene expression analysis is to identify a particular gene of interest, and then to study or characterize its expression profile in different hosts and/or treated tissues. For instance, based on the findings that during the early phases of the interaction between *P. infestans *and potato, the genes *ipiB *and *ipiO *are expressed at high levels, Pieterse et al. hypothesized that these genes played an important role in the early stages of the infection process [13]. Both genes were isolated and their expression studied in various host tissues and different host plants. The results showed that the expression of these genes was activated in compatible, incompatible and non-host interactions. In the case of *ipiO*, it was revealed that a motif on the promoter region functioned as a glucose repression element in yeast. This observation helped to generate hypotheses about its behavior in cultivars with different resistance levels. The authors concluded that perhaps a variable nutrient environment could trigger the expression of *ipiO *and *ipiB *depending on the host and/or the expressing tissue.

Most of the crucial *P. infestans *protein elicitors known to-date [14] have also been revealed by this approach. This is the case for the *Avr3a *avirulence gene, the first to be cloned from *P. infestans*. Subsequently, this gene was the subject of the first report of cell death suppression from a filamentous plant pathogen [15,16].

Differential expression of particular genes has also been used to study Systemic Acquired Resistance (SAR) and HR in challenged plants [17,18] to test, for instance, the correlation between the expression of basal SAR marker genes with resistance to *P. infestans *[19].

#### High throughput differential gene expression

This approach focuses in the identification of all the genes expressed in a cell under a particular condition. Since this approach allows us to differentiate clearly between the expression profiles of cells under different conditions, its application is of special interest in plant-pathogen interactions, allowing us to solve research questions such as: which genes are expressed in a compatible interaction that are not expressed in a compatible one? Or, are there any sub-regulations, positive or negative feed-backs, present in one case but not the other?

Different techniques such as DNA microarrays [20-23], serial analysis of gene expression [24,25] and differential display [26,27] have been used to study high throughput differential gene expression.

In the case of *P. infestans*, genes expressed in host cells challenged by this pathogen have been screened on compatible [28-30] and incompatible interactions [31-33], elucidating important issues about the mechanisms of interaction with its hosts.

For instance, gene regulation was revealed in a DNA microarray analysis of 7680 potato cDNA clones, representing approximately 5000 unique sequences expressed during a compatible interaction [30]. This work focused on the role of gene suppression in the compatible interaction, and its profile was obtained from microarray data evaluated at five time points. From this study, suppression of genes involved in the jasmonic acid (JA) defense pathway was revealed [34], as well as a severe down-regulation of the carbonic anhydrase (CA) gene, responsible for the reversible hydration of carbon dioxide to bicarbonate. Further analysis showed that CA was first down-regulated and then up-regulated during the incompatible interaction, clearly differentiating susceptibility from resistance, opening questions about the mechanisms that lead to its rapid suppression and the possibility of a connection between *CA *suppression and the overall down-regulation of the *JA *defense pathway.

Differential expression has also been studied on the pathogen side in *P. infestans *[35,21,23] and other *Phytophthora *species [21,36], revealing differential expression of e.g. the hsp70 and hsp90 genes, under distinct pathogen developmental stages and pathogenicity structures [37,36].

Although still fragmented, this approach provides a systemic view of the pathogenicity process, considering gene expression as a network and helping us to develop strategies to control or prevent the disease by manipulation of either the pathogen or the host.

### Structural and comparative genomics

Along with differential gene expression analysis, this is the most common modern approach to studying plant pathogen interactions, mostly due to the proteomic techniques as well as data mining and functional genomics tools available nowadays.

To date, one nuclear and six chloroplast genomes have been sequenced and two more nuclear genome sequencing projects are in progress in *Solanaceous *species (Additional file 1). On the pathogen side, five Oomycete genomes have been sequenced [11] and several studies at the genome scale have been carried out thanks to the availability of genomic information on these Oomycetes [38-40] and their hosts.

Therefore, the possibility of performing comparisons between different organisms at the sequence level [40] has allowed agronomically important resistance genes in potato to be isolated [41], pathogen avirulence genes [42] and gene families [10] to be identified, and novel proteins implicated in a given interaction to be identified [43]. For example, in the case of *S. tuberosum*, comparative analysis has revealed a physical co-localization between resistance loci in tomato, tobacco and pepper [44].

This approach has also revealed how two widely divergent microorganisms, *P. infestans *and the human malaria parasite *Plasmodium falciparum*, use equivalent host-targeting signals to deliver virulence and avirulence gene products into their hosts [45]. These products have been characterized by a particular protein motif, leading to the hypothesis of pathogenicity mechanisms conserved between both organisms [46]. This motif is the host-targeting (HT) signal of *P. falciparum*, centered on an RxLx core, revealed after the discovery of the RxLR host translocation motif of Oomycete effectors [47-49]. Owing to the availability of such data, it has been shown that although *Plasmodium *and *Phytophthora *are divergent eukaryotes, they share leader sequences, which suggests a conserved machinery for transport of effector proteins, a finding otherwise hard to achieve.

### Protein-protein interactions

One approach to study protein-protein interactions is by using yeast two hybrid screening, co-immunoprecipitation [50] or surface plasmon resonance. This is arguably the most important approach towards a broad understanding of any plant pathogen interaction. It enables some mechanisms for the suppression of host defense in several organisms, such as the fungal pathogen *Septoria lycopersici *[51] or the Oomycete *Phytophthora sojae *[52], to be revealed.

In the case of *P. infestans*, relevant host defense suppression molecules have been also identified by this approach, such as the extracellular protease inhibitors *EPI1 *[53], *EPI10 - *the first protease inhibitor reported in any plant-associated pathogen, which suppresses tomato defense by targeting - the *P69B *subtilisin-like serine protease [54], and the *EPIC *family of secreted proteins that target the extracellular cysteine protease PIP1 (*Phytophthora *Inhibited Protease 1) [55].

Protein-protein interactions play an important role in recognition between plant pathogens and their hosts. This recognition has been studied at two levels: recognition of the host by the pathogen and recognition of the pathogen by the host [56,57]. During an interaction, host resistance (*R*) and pathogen avirulence (*Avr*) proteins interact in a gene-for-gene manner. Proteins encoded by *R *alleles recognize the products of corresponding *Avr *alleles, thus triggering disease resistance. Using an association genetics approach [58], the *P. infestans Avr3a *effector was shown to be recognized in tomato cytoplasm by *R3a *(a member of the *R3 *complex locus on chromosome 11). *R3a *was isolated by positional cloning the same year [41].

Together, these and other studies [59,23], along with computational chemistry and/or computational modeling and prediction of protein-protein interactions [60], provide valuable information about the recognition mechanisms in *S. tuberosum *- *P. infestans R-Avr *interactions and could lead to the identification of metabolic and/or signaling pathways underlying incompatible interactions.

### Quantitative models in plant pathology

In cases where experimental data for a biological system start to accumulate, it is feasible and convenient to integrate all the information gathered into a quantitative model. This approach allows us to obtain a mathematical and networked framework for a descriptive model of the biological phenomenon [61]. This type of model strengthens the predictive capacity of future responses, for instance under different conditions, and it also helps to broaden our view of the potential interactions that could take place in any molecular reaction [62].

In order to capture time-dependent dynamic phenomena, a systems biology approach should allow us to integrate various ranges of spatial and temporal biological scales, as well as processing of different signals, genotypic variation and responses to external perturbations. As seen in the previous section, typical experiments describing the interaction between *P. infestans *and its hosts are clearly related to each of these characteristics.

Functional genomics and proteomic approaches produce the most suitable data for the development of a theoretical model [61]. For instance, microarray-based differential expression analysis evaluates expression patterns at different times [30], under different conditions [21,33] with host and pathogen genotypic variation. On the other hand, gene expression and host targeting of protease inhibitors work at different levels of signaling and at different spatial and temporal scales [54,53].

Data gathered from such plant-pathogen interaction approaches, along with the development of interaction, pathways and metabolism databases [63,64], as well as standardized systems biology languages [65,66] and *in silico *research platforms [67,68], have opened the door to modern computational model approaches at the molecular level in several organisms, including Oomycetes.

Predominantly, phytopathologists have used computational and quantitative modeling approaches to describe the temporal dynamics of plant diseases. Consistently, the bulk of the literature written in this field has been focused on the epidemiology of the disease, so research on the modeling of plant-pathogen molecular interactions is under-represented.

### Quantitative modeling of plant-pathogen epidemiology

#### Deterministic approaches

In 1969, Waggoner and Horsfall published Epidem, the first computer simulation of a plant disease [69]. Epidem was mainly a simulator of potato and tomato blights. Since then, models used in the plant-pathogen field have often belonged to the family of logistic equations.

The fundamental logistic model was proposed in 1963 by VanderPlank [70,71] and it describes the rate at which a disease spreads over time (Table 1).

**Table 1:** Solanaceous genome projects.

Species	Genome	Status	reference
*Nicotianatabacum*	mitochondrion	Finished	[106]
*Nicotianatomentosiformis*	chloroplast	Finished	[107]
*Solanum tuberosum*	chloroplast	Finished	[108]
*Solanum bulbocastanum*	chloroplast	Finished	[109]
*Solanum lycopersicum*	chloroplast	Finished	[110]
*Nicotianasylvestris*	chloroplast	Finished	[111]
*Atropa belladonna*	chloroplast	Finished	[112]
*Solanum tuberosum*	Nuclear	In progress	12984*
*Solanum lycopersicum*	Nuclear	In progress	9509*

*NCBI's genome project identification number.

In this model [71], *y*_*t *_is the proportion of diseased tissue (severity) at time *t *and *λ *is the rate of change of diseased tissue unit in a given unit time. The term (1-*y*_*t*_) indicates that new infections occur only in non-infected tissue. The slope of the disease curve depends on the infection rate (*λ*) and the inoculum *y*_*t*_. At a higher infection rate, the curve rise more steeply.

However, this model assumes that a lesion always remains infectious and also neglects the lag between the time at which an infection occurs and the time it becomes infectious (latent period). As such, the so-called generalized model considers both a latent period (*p *> 0) and an infectious period (*i*) [71,72] (Table 1). These values can range from *p *< 7, *i*<65 days in *Puccinia recondita *[73] to *p *< 2, *i*<8 days in *P. infestan*s [74].

Other relevant characteristics of a plant disease are its spatial pattern and the arrangement of disease entities (i.e., spores). The spatial patterns are influenced by dispersal of disease entities [75]. In cases where spore dispersal is not carried out directly from infected tissue but by environmental factors such as wind, a model assuming a constant source of inoculum, such as a monomolecular model, is more appropriate [71].

In this case the infected tissue is not part of the source, so the shape of the disease curve depends solely on the rate of infection. In the case of *Phytophthora*, five potential mechanisms of dispersal have been described for some major species [75]; for *P. infestans, P. cinnamoni *and *P. syringae*, a real mechanism of dispersal could be represented correctly by this model.

Some other models have been derived from the general logistic model. For instance, the Gompertz model is similar to the general logistic one and can be seen as a logarithmic form of it. When different data sets are compared, it is appropriate to use a model that allows us to make such comparisons; for those cases a Weibull model should be considered [76].

The spread of disease has also been modeled [77,78]. Since the early 1980s, the epidemic wave velocity of *P. infestans *has been measured by several means [79-82].

Although widely used, deterministic models do not represent the underlying biological process in a proper way. Spore germination is a good example of a stochastic process; for instance, examination of a single spore will reveal stochastic behavior, which can only be inferred by the examination of a significant number of units. Thus, in these cases, the process under study is better described by a probability function [2].

#### Stochastic approaches

Stochastic modeling of epidemics has been studied since the early 1960s. Most of the stochastic approaches carried out at that time were also concerned with the progress of the infection over time, represented by the so-called general stochastic epidemic model [83]:

Where (*τ*, *τ *+ *δ*_*τ*_) is the time interval. Here *I*(*τ*) represents the number of infectives, *S*(*τ*) the number of susceptibles and *R*(*τ*) the number of removals at time *τ *≥ 0. The removal of infected tissue is also considered probabilistic and it will occur with the following probability [83]:

in the same time interval, where *γ *> 0; *χ *= 0, 1, ..., *N*,. Since a given removal does not depend on previous ones, a removal is considered independent [83]:

Transition probabilities are given as [83]:

In non-stochastic models, stochasticity can be approached by adding randomness to state variables. For instance, Vanderplank's model was used in the description of the zucchini yellow mosaic virus disease [84]. In this case, stochasticity was achieved by adding a "*brownian motion term to the growth rate parameter*". As the authors stated, a significant difference between a stochastic and a deterministic version of the same model can be seen only if large data sets are employed. This observation could explain why in recent years, when biological data acquisition has grown faster than ever, the use of stochastic models has become more popular.

Stochastic modeling in plant pathology has also been applied to processes at different levels of biological organization, such as at the organ level, crops [85], spatial patterns, evolution [2] and aerial spread [86]. For instance, the spatial spread of disease in race-specific and race-nonspecific cultivar mixtures was studied using a *spatially explicit stochastic model *[87]. This model was based on the assumption that disease can be significantly higher in monocultures than in cultivar mixtures and it only considered stochastic variation of spore dispersal at constant sporulation rate, although there exist many other sources of stochastic variation (such as genotypic variation) [2].

No matter whether they are stochastic or deterministic, the models described above have been focused on higher scales of plant pathogen interactions, such as the population, organ or ecological level. Nevertheless, in any plant-pathogen disease, the molecular level of the interaction (i.e., protein-protein, protein-DNA, regulatory and metabolic network regulation) is intrinsically involved and surely accounts for much of the variation observed at other levels. Therefore, genetic processes in an organism can be seen as networks that *bridge the gap between genotype and phenotype *[88]. A good example of this situation can be found in the collective behavior of bacteria in Quorum Sensing (QS) mechanisms.

QS is a common strategy used by several plant-pathogenic bacteria to assess local population density and/or physical confinement. In a recent publication, a model describing the Ti plasmid quorum-sensing gene network was constructed [89]. It was shown that it could operate as an "on-off" gene expression switch that is sensitive to the environment, allowing the question about how bacteria really behave or respond to be answered in QS.

Although this topic is absent in some plant-pathogen organisms, such as *P. infestans*, the characteristics of quantitative modeling of molecular mechanisms could elucidate several questions in phytopathology.

### *In silico *modeling of plant-pathogen molecular interactions

Plants resist pathogen attacks by shifting their defense mechanisms, as reflected in quantitative and kinetics enhancements [62]. The mechanism that controls host defense activation consists of a highly interconnected network, in which host defense genes interact with each other as well as with effector proteins present in the cell [90-92]. The availability of high-throughput gene expression and proteomics data has generated an unprecedented opportunity for comprehensive study of these types of biological networks [89,93].

Since an important phase in host-pathogen interactions involves protein-protein recognition [94,91], efforts to elucidate networks of such interactions are of special interest in phytopathology. For example, a whole-genome computational strategy to infer protein interactions was applied to ten pathogens, including species of *Mycobacterium, Apicomplexa and Kinetoplastida *[91]. This work started with the identification of pairs of matching proteins known to interact between the host and the pathogen, and by assessing the likelihood of this interaction by means of structural modeling, expression properties and subcellular location. As a result, an enriched candidate set of proteins is obtained, suitable for experimental study.

With the current genome sequence information for several *Phytophthora *genomes (Additional file 1) and those under sequencing [11,95], this approach could be applicable to a *Phytophthora-Solanaceae *model and thus enhance our limited knowledge about the molecular interactions in these genera.

**Figure 1:**
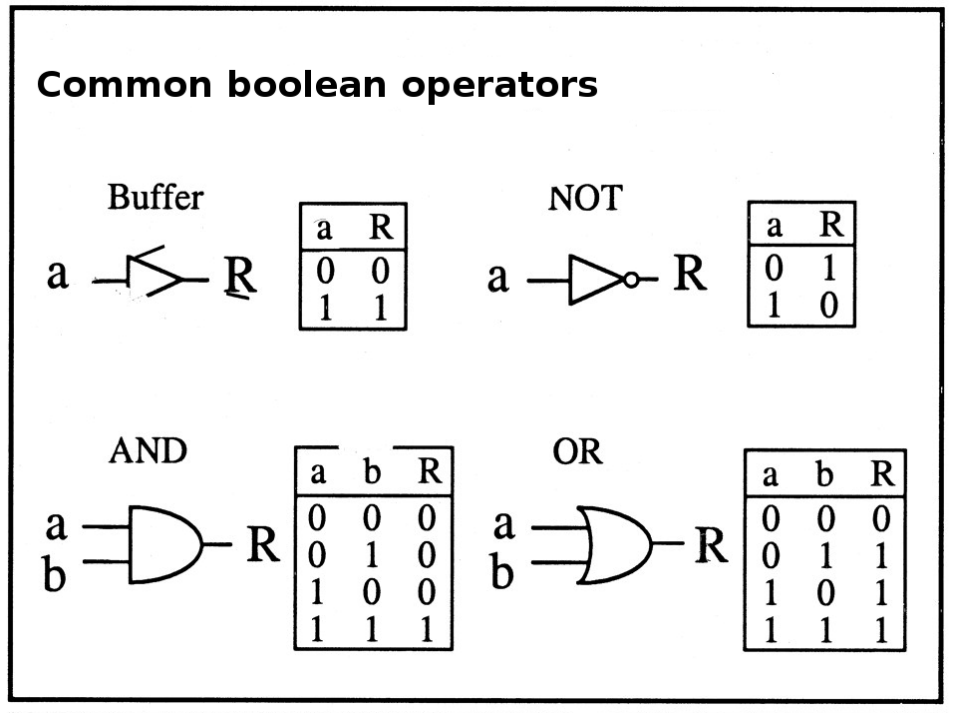
**Boolean formalism**. Adapted from [98] The most frequent types of boolean operators are the buffer, NOT, AND and OR gates. Tables adjacent to each of these gates are known as "true" tables, where "a" and "b" represent the input (or stimuli) and R the output (or response).

Another good theoretical framework to start working with is a *space of interconnected operators *such as a boolean network (Figure 1). Boolean networks present some advantages when compared to similar strategies such as hidden Markov models [96,97]. For instance, it is possible to perform a simulation while "avoiding the statistical basis around them, provides the option to perform simpler computational simulations, insert additional regulators or quantitative and biochemical data parameters into the model when available" [98].

### Towards a boolean description of the *P. infestans - Solanum tuberosum *interaction

**Figure 2:**
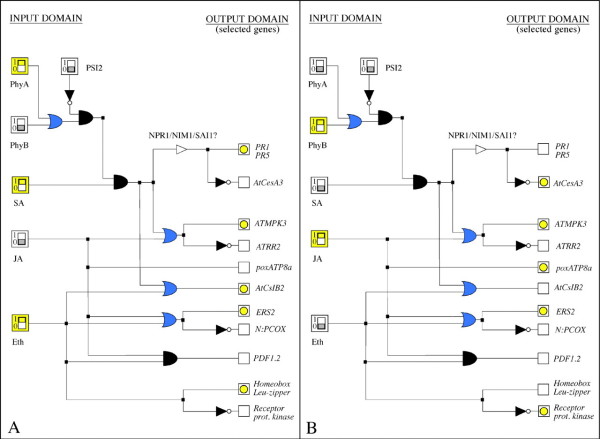
**Boolean representation of a signaling network**. Adapted from [98] Boolean representation of the signal transduction network controlling the plant's defense response against pathogens in *Arabidopsis thaliana*, represented by a series of output genes selected from microarray data. The activated switches are represented in yellow. Diode symbols in yellow indicate the induced genes. Empty squares correspond to no significant expression. A and B represent two of the various possible outputs given the input.

Although clustering analysis can be used to infer gene function from expression data, the detailed interaction between genes within or between clusters cannot be deduced by this approach [99]. In order to deduce such interactions, data from differential expression analysis can be represented in a boolean formalism. This representation can be achieved in a typical boolean binary form, where repression and/or induction of a given gene can be expressed by an *on *or *off *switch and thus translated into a network structure and simulated by computational analysis. This approach has been successfully implemented in the simulation of plant defense signaling networks in *Arabidopsis thaliana *in response to different treatments with salicylic acid, jasmonic acidand ethylene [98] (figure 2). For example, genes up-regulated to the same level in both treatments can be expressed by an OR operator, as in the case of *phyA *and *PhyB *in figure 2, thus leading to different possible initial states (input domains) as represented by panels A and B in the same figure. Data from differential expression analysis can also be represented by three possible boolean states (Table 2). This approach has been successfully used in the inference of gene regulatory networks [100] where "-1" was also introduced to address the negative interaction between components in the network. Experimental information on the compatible interaction between *P. infestans *and *S. tuberosum *is being approached by our laboratory using a similar strategy, in order to hypothesize the network space of carbonic anhydrase in this interaction.

**Table 2:** Boolean representation of defense-related genes expressed during a compatible interaction between *P. infestans *and *S. tuberosum*.

Gene name	6 h		12 h		24 h		48 h		72 h	
Carbonic anhydrase	***0.6***	**-*1***	***0.6***	**-1**	***0.5***	**-1**	***0.3***	**-1**	***0.3***	**-1**
Proteinase inhibitor II	0.9	0	1.1	0	***0.5***	**-1**	***0.5***	**-1**	***0.4***	**-1**
Peroxiredoxin	1.0	0	0.8	0	***0.7***	**-1**	***0.7***	**-1**	***0.7***	**-1**
2-Cys peroxiredosin	1.0	0	0.9	0	***0.6***	**-1**	***0.6***	**-1**	***0.5***	**-1**
Proteinase inhibitor I	1.0	0	0.9	0	***0.6***	**-1**	0.9	0	0.6	0
Superoxide dismutase	0.9	0	0.8	0	0.8	0	***0.7***	**-1**	***0.7***	**-1**
Peroxidase	0.8	0	0.9	0	0.9	0	0.8	0	***0.6***	**-1**
Aspartic proteinase inhibitor	**1.7**	**1**	1.0	0	1.2	0	1.1	0	***0.7***	**-1**
Cystein proteinase inhibitor	**1.5**	**1**	**1.9**	**1**	1.1	0	1.2	0	***0.6***	**-1**
Cysteine protease	1.0	0	1.4	0	1.3	0	1.4	0	**2.0**	**1**
Peroxidase	1.2	0	1.3	0	1.3	0	1.4	0	**2.5**	**1**
Catechol oxidase	**1.5**	**1**	**2.4**	**1**	1.4	0	**1.8**	**1**	**2.5**	**1**
Catalase	1.4	0	**1.7**	**1**	**1.9**	**1**	**2.3**	**1**	**3.3**	**1**
Glutathione reductase	**1.8**	**1**	**1.8**	**1**	**1.6**	**1**	**2.3**	**1**	**3.0**	**1**

Adapted from [30]. Inductions, quantified as ratios greater than 1.5-fold, are represented in bold font. Repressions, quantified as ratios lower than 1.5-fold, are represented in bold and italic font. Grey columns contain the boolean representation for each gene expression level. Inductions here are represented by 1, repressions by -1 and non-significant expression by zero. These boolean values can further be formalized and simulated into a computational model.

This approach can also be used in systems lacking biological information, by gathering data common to other organisms or from related species. This possibility opens the door to implementation in other species of Oomycetes where lack of information is typical.

## Conclusion

The idea of the stochastic modeling of biological systems is not new, although traditionally, the mathematical frameworks used to represent and study these processes have been deterministic. This situation can be explained by taking into consideration the fact that quantitative and computational modeling usually require the availability of important computational resources. These resources increase proportionally with the number of variables involved in the model; then apart from restrictions on the availability of biological information included in the model, there also exist restrictions on the availability of computational resources to perform a given simulation. In recent years, computational resources have become less restrictive. Moreover, stochastic processes are probabilistic in nature and thus require the use of more data as confidence in calculations depends on them. More data means more calculations and interconnections between variables. Thus, the availability of computational resources and biological data has restricted the use of stochastic approaches for decades, not only in plant pathology, but in biological processes in general.

Any top level biological observation implies an underlying molecular process, which is not isolated from its environment. This situation has always been evident to plant pathologists, as reflected in the conversion of environmental factors such water or humidity into model variables. Epidemiological research will find molecular plant-pathogen interaction models an important tool for describing disease spread and dynamics from its roots. In turn, molecular plant pathogen interactions cannot be modeled in isolation from environmental variables, largely analyzed by deterministic approaches since the early 1960s. Therefore, in order to reflect the real biological phenomena, it is crucial to take this information into account when a molecular interaction is considered.

Biological information that implies a networked structure can be represented by a boolean formalism. This approach avoids the immediate necessity of chemical kinetics and the use of sets of equations (for example differential equations) to run a simulation. Thus, boolean networks are a viable and ideal strategy for the computational modeling of protein-protein interactions, metabolic networks and differential expression data available today for organisms for which molecular kinetic information is not available.

To date, differential gene expression data, protein-protein interaction and functional comparative analysis represent the only information available, not only for the majority of Oomycetes and their hosts but also for several other organisms. Here we argue that, due to the type of information available - although hidden Markov models, neural networks and flux balance analysis have recently been used - a boolean representation of plant-pathogen interactions between *P. infestans *and *S. tuberosum *is one of the most suitable approaches for computational modeling; an ongoing effort in our laboratory, based on microarray data for a compatible interaction between these organisms. Once available, boolean networks will allow kinetic information to be put back into the model and thus complement it with new information as it becomes available.

Quantitative representation and computational simulation of biological data is an important tool for understanding complex biological networks and interactions. To date, the availability of efficient algorithms, biological information and computational resources have opened the door to new insights into the analysis of such information. Bioinformatics, systems biology and its most representative tool, computational modeling, allow us to study complex plant pathogen interactions in a way unreachable to scientists two decades ago. Understanding of plant-pathogen interactions at the deterministic and stochastic, molecular and population levels requires a holistic approach, where any piece of available information is important. Today we are facing an integrative era of biological information, which approaches biological phenomena not from their individual parts but from their interactions. Without a doubt, this approach reflects biological reality in a more convenient and realistic way, but it also brings new challenges as well as the necessity for new tools, which cover not only the biological sciences field but also engineering and mathematics.

## Competing interests

The authors declare that they have no competing interests.

## Authors' contributions

AP and SR conceived the overall direction and major sections of the manuscript. All authors contributed to writing the manuscript.

## Supplementary Material

Additional file 1 Fundamental deterministic models used in plant pathology. Click here for file
