# Supplementary material for: Computational models in plant-pathogen interactions: the case of Phytophthora infestans
Source: Theor Biol Med Model. 2009 Nov 12;6:24. doi: 10.1186/1742-4682-6-24 (PMC2787490; doi:10.1186/1742-4682-6-24)
Supplement: Additional file 1 — Fundamental deterministic models used in plant pathology. [file 1742-4682-6-24-S1.pdf]

Additional file 1: Fundamental deterministic models used in plant pathology.

Name	Model	Citation
Mono molecular model	$\lambda(1-y_{\tau})$	[101]
Gompertz model	$\lambda y_{\tau} \ln\frac{1}{y_{\tau}}$	[102]
Weibull model	$\frac{\lambda}{\beta}\left(\frac{\tau-\alpha}{\beta}\right)^{\gamma-1}\exp\left[-\left(\frac{\tau-\alpha}{\beta}\right)^{\gamma}\right]$	[103]
Fundamental logistic model	$\lambda y_{\tau}(1-y_{\tau})$	[104]
Generalized model	$R(y_{\tau-p}-y_{\tau-p-i})(1-y_{\tau})$	[105]

Vanderplank's model assumes that latent period *(p)* has an effect on disease and that infected tissue duration *(i)* is infinite. Generalized model includes *P*, *i* and *R* as a corrected infection rate. In cases of such systemic infections or when a pathogen itself is transported by environmental factors, the corrected infection rate is assumed as in Vanderplank's original logistic model, since *P* can be assumed as *0* and *i* as infinite. Several logistic models have been derived from Vanderplank, generalized and monomolecular models, such Gompertz and Weibull models.
